# Carbon dioxide inhibits UVB-induced inflammatory response by activating the proton-sensing receptor, GPR65, in human keratinocytes

**DOI:** 10.1038/s41598-020-79519-0

**Published:** 2021-01-11

**Authors:** Keimon Sayama, Katsuyuki Yuki, Keiichi Sugata, Satoko Fukagawa, Tetsuji Yamamoto, Shigaku Ikeda, Takatoshi Murase

**Affiliations:** 1 Biological Science Research, Kao Corporation, 2606, Akabane, Ichikai-machi, Haga-gun, Tochigi 321-3497 Japan; 2 Department of Dermatology and Allergology, Juntendo University Graduate School of Medicine, Tokyo, Japan

**Keywords:** Skin manifestations, Disease prevention, Inflammation

## Abstract

Carbon dioxide (CO_2_) is the predominant gas molecule emitted during aerobic respiration. Although CO_2_ can improve blood circulation in the skin via its vasodilatory effects, its effects on skin inflammation remain unclear. The present study aimed to examine the anti-inflammatory effects of CO_2_ in human keratinocytes and skin. Keratinocytes were cultured under 15% CO_2_, irradiated with ultraviolet B (UVB), and their inflammatory cytokine production was analyzed. Using multiphoton laser microscopy, the effect of CO_2_ on pH was observed by loading a three-dimensional (3D)-cultured epidermis with a high-CO_2_ concentration formulation. Finally, the effect of CO_2_ on UVB-induced erythema was confirmed. CO_2_ suppressed the UVB-induced production of tumor necrosis factor-α (TNFα) and interleukin-6 (IL-6) in keratinocytes and the 3D epidermis. Correcting medium acidification with NaOH inhibited the CO_2_-induced suppression of TNFα and IL-6 expression in keratinocytes. Moreover, the knockdown of H^+^-sensing G protein-coupled receptor 65 inhibited the CO_2_-induced suppression of inflammatory cytokine expression and NF-κB activation and reduced CO_2_-induced cyclic adenosine monophosphate production. Furthermore, the high-CO_2_ concentration formulation suppressed UVB-induced erythema in human skin. Hence, CO_2_ suppresses skin inflammation and can be employed as a potential therapeutic agent in restoring skin immune homeostasis.

## Introduction

The skin is the largest organ of the human body. It acts as a protective covering while serving as a barrier separating the body from the external environment. The stratum corneum and tight junctions in the granular layers prevent the entry of external stimuli such as antigens, microorganisms, and ultraviolet (UV) radiation from the external environment, as well as water evaporation from the internal environment^1^. When antigens and pathogens enter the skin through these physical barriers, resident/infiltrated immune cells induce an immune response to eliminate them. Epidermal keratinocytes, which are responsible for building a physical barrier, play an important role in skin immunity and cooperate with immune cells by producing various growth factors, cytokines, and chemokines in response to external stimuli^2^. In addition, UV radiation from sunlight is a major stress source for keratinocytes and affects various biological functions including the nervous system and endocrine system through the skin^3^. UV-induced oxidative stress leads to mitochondrial dysfunction and activation of the nuclear factor kappa B (NF-κB) pathway, a major inflammatory response pathway, resulting in cell death^4–6^. On the other hand, there are multiple UV-responsive stress proteins in keratinocytes, such as the NF-E2 related factor (NRF) family, which play a role in suppressing oxidative stress to prevent excessive cell death^7–9^. Consequently, the skin functions as an immunological organ; however, excessive immune responses can lead to chronic skin inflammation and further to inflammatory skin diseases such as atopic dermatitis and psoriasis^10^. These diseases are difficult to cure and are accompanied by physical and mental stress due to skin symptoms that significantly reduce the quality of life, such as pruritus, redness, and lichenification. Therefore, the daily prevention of excessive immune responses is vital for maintaining skin immune homeostasis.

Carbon dioxide (CO_2_) is one of the principal gas molecules responsible for aerobic respiration. In the cell, CO_2_, a by-product of oxidative metabolism in the tricarboxylic acid (TCA) cycle, passively diffuses through the body and is expelled via the lungs by red blood cells. In mammals, neurons in the brainstem and the peripheral carotid body detect blood CO_2_ pressure, usually maintained at approximately 40 mmHg^11^. This CO_2_-sensing system also exists in flies and nematodes^12,13^, implying that CO_2_ homeostasis is critical for the survival of various species.

Some gas molecules have been shown to function as signaling mediators called ‘gasotransmitters’^14^. Among them, hydrogen sulfide and nitric monoxide are well-known and are involved in physiological functions in the skin, such as vasodilation, cell proliferation, apoptosis, and inflammation^15,16^. These facts suggest that gasotransmitters play an important role in maintaining skin homeostasis. Recent studies have reported that CO_2_ improves alveolar damage in rat models and patients with acute respiratory distress syndrome^17–19^. In addition, CO_2_ has been shown to suppress lipopolysaccharide (LPS)-induced inflammatory responses in several blood cell types and lung-derived cell lines^20–22^. Consequently, this points to CO_2_ not only as a by-product of aerobic respiration but also to its role as a gasotransmitter in suppressing inflammatory responses. In the skin, CO_2_ has been shown to improve wound healing and seasonal barrier dysfunction^23–25^; inflammation is known to be strongly involved in skin wound healing and barrier functions as well as the circulatory system. The wound-healing process consists of three phases: the inflammatory phase, proliferative phase, and stable phase; this process may be delayed when an excessive inflammatory response is triggered during the inflammatory phase^26^. Various cytokines secreted from keratinocytes, fibroblasts, and inflammatory cells are critical factors that influence skin barrier function and keratinocyte differentiation^27^; however, little is known about the relationship between CO_2_ and skin inflammation.

In the present study, the anti-inflammatory mechanism of CO_2_ was elucidated using a UVB-irradiated human keratinocyte model, focusing on the CO_2_-induced decrease in extracellular pH. In addition, the effect of CO_2_ on UV-induced erythema was evaluated in human skin.

## Results

### CO_2_ inhibited UVB-induced cytokine expression in HEKn and the 3D epidermis

**Figure 1:**
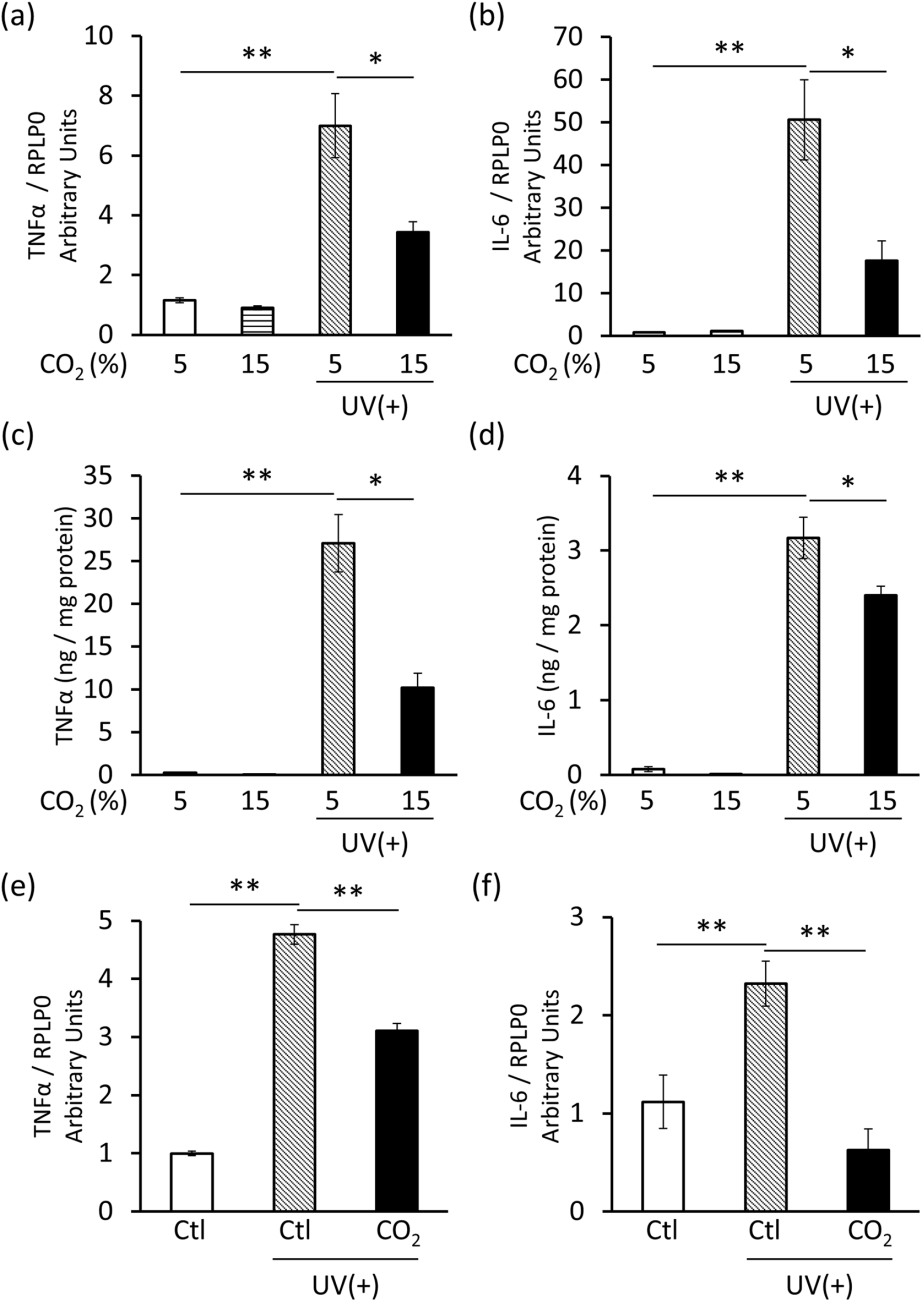
CO_2_ inhibits UVB-induced cytokine expression in HEKn and the 3D epidermis. (**a**,**b**) HEKn were incubated in 5 or 15% CO_2_ for 24 h and then irradiated with 20 mJ/cm^2^ of UVB. Total RNA was isolated 8 h later and qRT-PCR was performed to detect TNFα and IL-6 mRNA expression (*n* = 3, * *P* < 0.05, ** *P* < 0.01 vs. 5% CO_2_ UV(+), Dunnett’s test). (**c**,**d**) The culture supernatant was collected 24 h after UVB irradiation and ELISA was performed to measure TNFα and IL-6 concentration (*n* = 3, *P < 0.05, **P < 0.01 vs. 5% CO_2_ UV(+), Dunnett’s test). (**e**,**f**) CO_2_-free (Ctl) and high-CO_2_ concentration (CO_2_) formulations were applied to the surface of the 3D epidermis for 12 h and then exposed to UVB. Total RNA was isolated 8 h later, and qRT-PCR was performed to detect TNFα and IL-6 mRNA expression (*n* = 3, **P < 0.01 vs. Ctl UV(+), Dunnett’s test).

Human epidermal keratinocytes isolated from neonatal foreskin (HEKn) cultured in 15% CO_2_ displayed a significantly lower UVB-induced increase in TNFα and IL-6 mRNA expression (Fig. 1a,b) alongside significantly lower TNFα and IL-6 protein levels in the culture supernatant (Fig. 1c,d). In addition, the 3D epidermis treated with the high-CO_2_ concentration formulation (CO_2_) displayed a significantly lower UVB-induced increase in TNFα and IL-6 mRNA expression than the 3D epidermis treated with the control (Ctl) formulation (Fig. 1e,f). Taken together, these data indicate that CO_2_ suppresses the production of UVB-induced inflammatory cytokines in the epidermis.

### CO_2_ induced extracellular acidification, which produced anti-inflammatory effects

**Figure 2:**
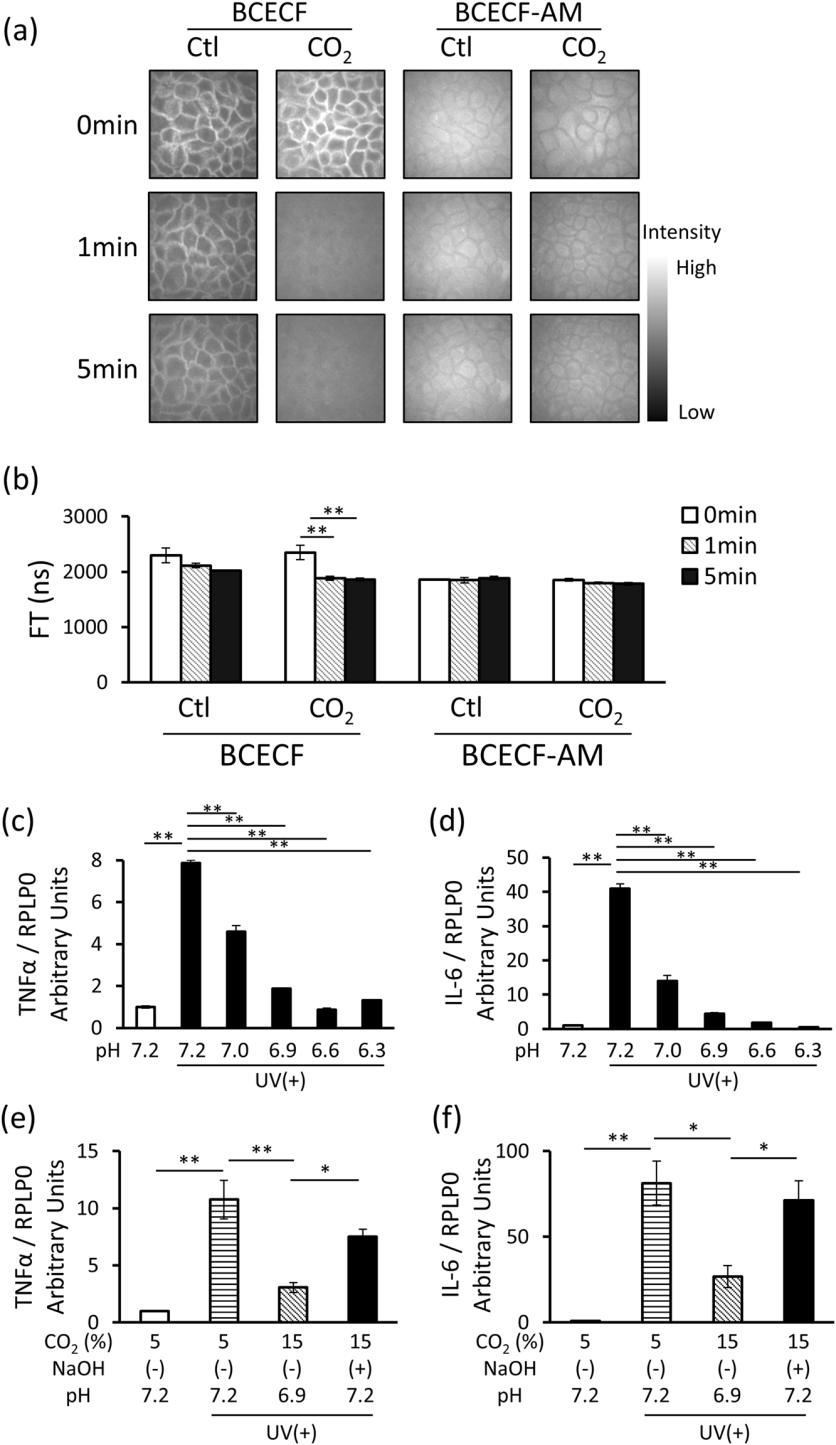
CO_2_ induces extracellular acidification, generating anti-inflammatory effects. (**a**) CO_2_-free (Ctl) and high-CO_2_ concentration (CO_2_) formulations were applied to the surface of the 3D epidermis after exposure to BCECF or BCECF-AM. Intra-epidermal pH was visualized by multiphoton laser microscopy (DermaInspect). Fluorescent images were obtained 0, 1, and 5 min after formulation application. The images shown are representative of three experiments with similar results. (**b**) Representative fluorescence lifetime was calculated from the fluorescent images using SPC Image 2.9.4 (*n* = 3, **P < 0.01 vs. CO_2_ 0 min, Dunnett’s test). (**c**,**d**) HEKn were maintained at the indicated pH in an HCl-supplemented medium for 24 h and then irradiated with 20 mJ/cm^2^ of UVB. Total RNA was isolated 8 h later, and qRT-PCR was performed to detect TNFα and IL-6 mRNA expression (*n* = 3, **P < 0.01 vs. pH 7.2 UV(+), Dunnett’s test). (**e**,**f**) HEKn were incubated in 5 or 15% CO_2_ for 24 h and then irradiated with 20 mJ/cm^2^ of UVB. The low pH (6.9) induced by 15% CO_2_ was adjusted to pH 7.2 with NaOH. Total RNA was isolated 8 h later and qRT-PCR was performed to detect TNFα and IL-6 mRNA expression (*n* = 3, *P < 0.05, **P < 0.01, Tukey–Kramer test).

pH changes in the 3D epidermis were visualized using the pH-dependent fluorescent indicators, BCECF and BCECF-AM. BCECF can selectively visualize extracellular pH due to its membrane impermeability, whereas BCECF-AM can selectively visualize intracellular pH as it is hydrolyzed into membrane-impermeable BCECF by cytosolic esterase. In this study, we found that extracellular BCECF-derived fluorescence was attenuated when the high-CO_2_ concentration formulation was applied to the surface of the 3D epidermis (Fig. 2a) and the extracellular BCECF-derived representative FT decreased significantly, suggesting that CO_2_ administration caused extracellular acidification (Fig. 2b). In the HEKn, UVB-induced increases in TNFα and IL-6 mRNA expression were significantly suppressed depending on the acidification of the culture medium (Fig. 2c,d). Moreover, when the pH of the culture medium was neutralized using NaOH under 15% CO_2_ condition, the CO_2_-induced suppression of TNFα and IL-6 mRNA expression was reduced (Fig. 2e,f). Thus, these results indicate that CO_2_-induced extracellular acidification may suppress UVB-induced inflammatory cytokine expression.

### CO_2_-activated GPR65 signaling suppressed UVB-induced inflammation by inhibiting NF-κB activation in HEKn

**Figure 3:**
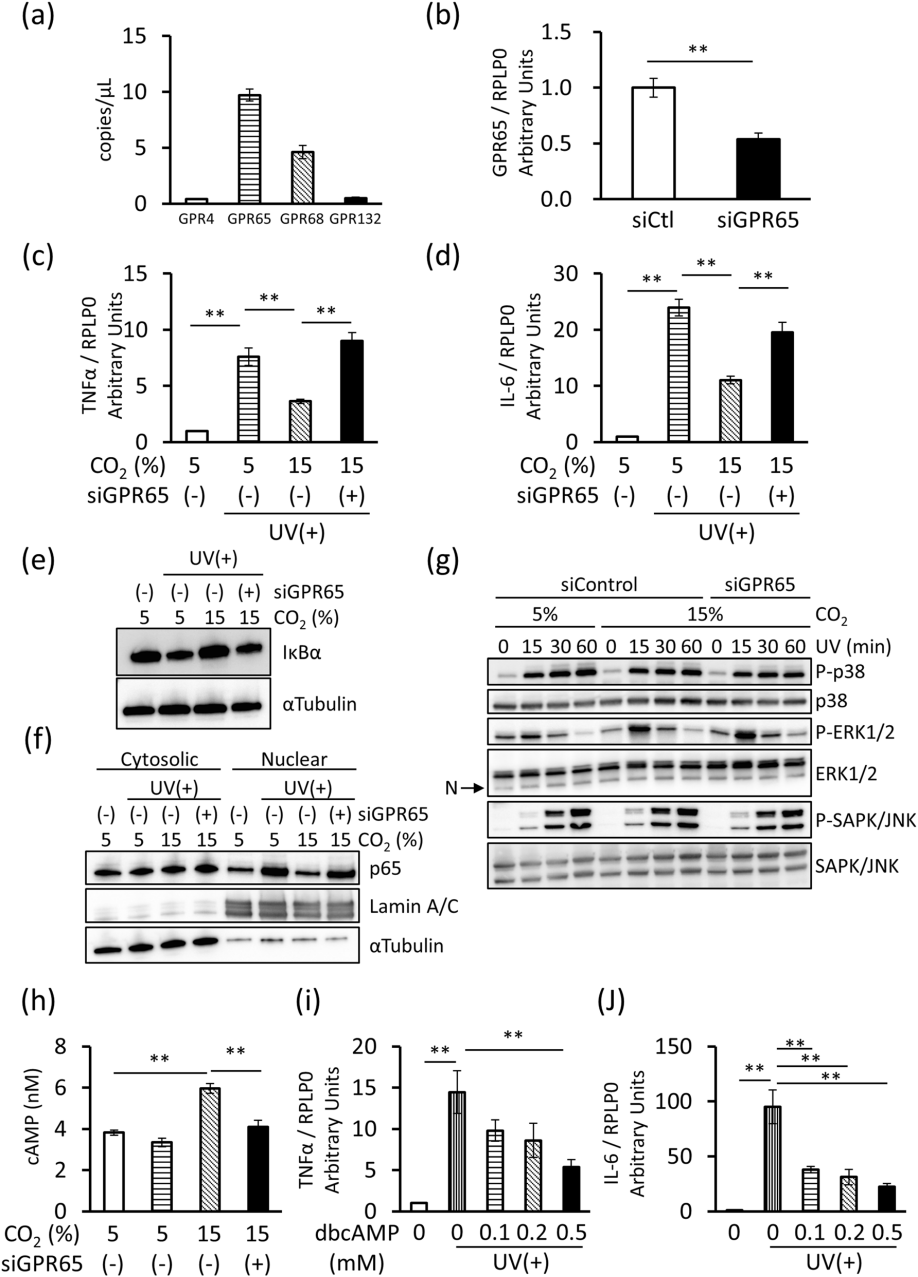
CO_2_-activated GPR65 signaling suppresses UVB-induced inflammation by inhibiting NF-κB activation in HEKn. (**a**) Digital PCR was performed to determine the mRNA expression of proton sensing GPCRs in HEKn (*n* = 3). (**b**) The cells were transfected with control non-target siRNA (siCtl) or specific siRNA against GPR65 (siGPR65). Total RNA was isolated 48 h later and qRT-PCR was performed to detect GPR65 mRNA expression (*n* = 3, **P < 0.01, unpaired Student’s *t* test). (**c**,**d**) The transfected cells were incubated in 5 or 15% CO_2_ for 24 h and then irradiated with 20 mJ/cm^2^ of UVB. Total RNA was isolated after 8 h, and qRT-PCR was performed to detect TNFα and IL-6 mRNA expression (*n* = 3, **P < 0.01, Tukey–Kramer test). (**e**) The cells were transiently transfected with siCtl or siGPR65, incubated in 5 or 15% CO_2_ for 24 h, and irradiated with 20 mJ/cm^2^ of UVB. Whole cell extracts were prepared 8 h later, and I-κBα and α-tubulin levels were assessed by western blot analysis. (**f**) Cytoplasmic and nuclear extracts were prepared 8 h after UVB irradiation, and p65, laminin A/C, and α-tubulin levels were assessed by western blot analysis. (**g**) Whole cell extracts were prepared 0, 15, 30, and 60 min after UVB irradiation and P-p38, p38, P-Erk1/2, Erk1/2, P-SAPK/JNK, and SAPK/JNK levels were assessed by western blot analysis. ‘N’ on the left indicates a non-specific band. Luminescent signal images are shown cropped and full-length blots/gels are presented in Supplementary Figures 1–6. (**h**) The cells were transiently transfected with siCtl or siGPR65. After 48 h, the cells were cultured in 5 or 15% CO_2_ for 24 h with 0.5 mM IBMX, and then intracellular cAMP was measured (*n* = 3, **P < 0.01 vs. CO_2_ 15% siGPR65(−), Dunnett’s test). (**i**,**j**) The cells were incubated with 0, 0.1, 0.2, or 0.5 mM of dibutyryl cAMP (dbcAMP) for 24 h and then irradiated with 20 mJ/cm^2^ of UVB. Total RNA was isolated 8 h later, and qRT-PCR was performed to detect TNFα and IL-6 mRNA expression (*n* = 3, **P < 0.01 vs. dbcAMP 0 mM UV(+), Dunnett’s test).

Extracellular pH changes are detected by H^+^-sensing GPCRs^28^. It was determined that GPR65 mRNA was highly expressed in HEKn compared to other H^+^-sensing GPCRs (Fig. 3a). Therefore, GPR65 was knocked down in HEKn using GPR65-specific siRNA to clarify its role in CO_2_-induced anti-inflammatory effects (Fig. 3b). GPR65 knockdown significantly inhibited the CO_2_-induced downregulation of TNFα and IL-6 mRNA expression (Fig. 3c,d); furthermore, it diminished the CO_2_-induced suppression of inhibitor-κBα (I-κBα) degradation and p65 nuclear translocation caused by UVB irradiation (Fig. 3e,f). However, no difference was observed in the UVB-induced phosphorylation of p38, extracellular signal-regulated 1/2 (ERK1/2), or c-Jun N-terminal kinase (JNK) following 15% CO_2_ incubation or GPR65 knockdown (Fig. 3g). Culture under 15% CO_2_ significantly increased the intracellular cAMP concentration; however, this CO_2_-induced increase was diminished by GPR65 knockdown (Fig. 3h). Moreover, when dibutyryl cAMP, a cAMP analog, was added to clarify the anti-inflammatory effect of cAMP in HEKn, UVB-induced TNFα and IL-6 mRNA expressions were significantly suppressed in a concentration-dependent manner (Fig. 3i,j). Taken together, these results indicate that CO_2_-induced extracellular acidification activates GPR65, increases intracellular cAMP concentration, and inhibits UVB-induced NF-κB activation.

### CO_2_ inhibited UVB-induced erythema in human skin

**Figure 4:**
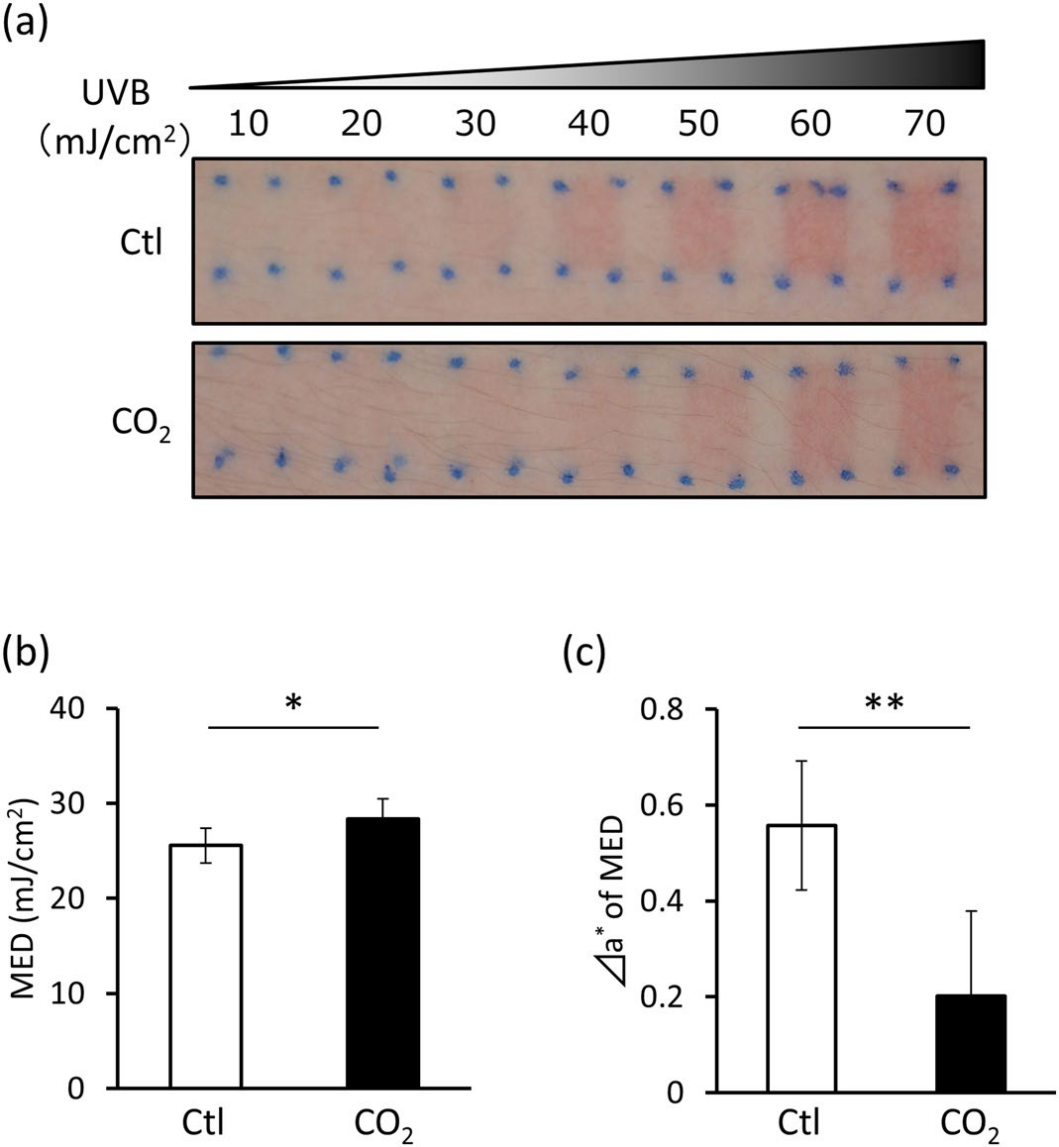
CO_2_ inhibits UVB-induced erythema in human skin. CO_2_-free (Ctl) and high-CO_2_ concentration (CO_2_) formulations were applied to the skin of the inner upper left arm twice daily for 2 weeks and then exposed to UVB. The next day, (**a**) UVB-irradiated skin images were obtained. The images shown are representative of the nine volunteers. (**b**) MED and (**c**) color-difference (Δ a* of MED) were assessed (*n* = 9, *P < 0.05, **P < 0.01, paired Student’s *t* test).

To clarify the anti-inflammatory effects of CO_2_ on human skin, control and high-CO_2_ concentration formulations were applied to the skin on the inner upper arm of nine men, followed by UVB irradiation. UVB-induced skin erythema formation and MED were suppressed in the skin area where CO_2_ had been applied (Fig. 4a,b). In addition, the Δa* value (obtained by subtracting the a* value of the non-irradiated site from that of the site irradiated with 1MED UVB) was significantly lower in the skin area where CO_2_ had been applied (Fig. 4c). Thus, these results indicate that CO_2_ may exert anti-inflammatory effects in human skin.

## Discussion

UV radiation emitted from the sun induces skin inflammation, which causes a wide range of skin symptoms from spots and wrinkles to dermatitis and skin cancer. In the present study, the anti-inflammatory effects of CO_2_ were examined using a UVB-induced inflammation model. It was found that CO_2_ suppressed TNFα and IL-6 production in human keratinocytes and the 3D epidermis and attenuated UVB-induced erythema formation in human skin. Since inflammatory cytokines such as TNFα and IL-6 are known to play key roles in skin inflammation^29,30^, CO_2_ may reduce UV-induced inflammation by suppressing their production.

The percutaneous administration of CO_2_ has been shown to increase blood flow in the skin by reducing vascular smooth muscle tension under the epidermis^31,32^, signifying that CO_2_ has relatively high transdermal permeability. Therefore, CO_2_ may permeate through the stratum corneum and react with H_2_O in the interstitial fluid to produce H^+^, resulting in mild extracellular acidification. Consequently, the role of extracellular pH in the anti-inflammatory effects of CO_2_ was investigated. In keratinocytes, the CO_2_-induced suppression of TNFα and IL-6 expression was dependent on the pH of the culture medium, indicating that extracellular pH exerts important effects on skin inflammation. Interestingly, it has been reported that UV irradiation induces intracellular pH reduction and cell death in keratinocytes^33^. These findings suggest that intracellular and extracellular pH changes exert different physiological effects. In the skin, the pH of the stratum corneum is known to play an important role in various pathological conditions. It is usually maintained in an acidic range of 4.1–5.8; however, it registers an increase in inflammatory skin diseases^34^. Previous studies have shown that the disturbance of pH homeostasis disrupts various skin functions such as the antimicrobial response, skin barrier action, and inflammation^35–37^. Thus, the topical application of CO_2_ may improve the barrier and antimicrobial functions of the stratum corneum and suppress excessive inflammatory responses in the epidermis via CO_2_-induced acidification.

Cells detect extracellular pH via acid-sensing ion channels and H^+^-sensing GPCRs^28,38^. Although Na^+^/H^+^ exchanger 1 (NHE1) has been reported to sense extracellular pH changes in the skin^39,40^, the biological sensors linking pH changes to skin inflammatory responses have not yet been elucidated. In the present study, it was demonstrated for the first time that GPR65, an H^+^-sensing GPCR, may detect CO_2_-induced extracellular acidification and exert anti-inflammatory effects in keratinocytes. GPR65 has been reported to act as a psychosine receptor^41^; however, it has also been found to act as an H^+^ sensor^42–44^, whose activation induces cAMP production via Gα_s_ signaling. In the present study, GPR65 knockdown in keratinocytes suppressed CO_2_-induced cAMP production, while dibutyryl cAMP, a cAMP analog, inhibited TNFα and IL-6 expressions. These results indicate that CO_2_-induced GPR65/cAMP signaling plays an important role in suppressing inflammation. There are various types of Gα subunits (Gα_s_, Gα_i_, Gα_q_, and Gα_12_), each transmitting different cellular signals^45^; however, it remains unclear whether GPR65 activates other Gα proteins.

UV irradiation is known to induce inflammatory responses by MAPK and NF-κB^46^. In this study, CO_2_ did not affect the phosphorylation of p38, ERK1/2, or JNK, implying that CO_2_ does not exert its anti-inflammatory effects via the MAPK pathway. However, CO_2_ did suppress UVB-induced I-κBα degradation and p65 nuclear translocation, while GPR65 knockdown opposed the CO_2_-induced suppression of NF-κB activation. Based on these results, it was hypothesized that CO_2_ exerts its anti-inflammatory effects by activating GPR65 and following suppression of the NF-κB pathway. While this finding is partly consistent with a previous work by Cummins et al.^47^, other studies have shown that CO_2_ does not affect I-κBα degradation in LPS-sensitized THP-1 cells and macrophages^22^. These conflicting results may be due to different NF-κB activation mechanisms since it was recently reported that there is a third UV-dependent NF-κB activation pathway in addition to the canonical and non-canonical pathways^48^. In this pathway, I-κBα is translocated into the nucleus by UV irradiation without phosphorylation, where it is degraded by forming a complex with β-Transducin repeat Containing Protein (β-TrCP), a subunit of the ubiquitin-protein ligase complex, using IKKβ as a scaffold. Therefore, GPR65 activation may affect this UV-dependent NF-κB pathway; however, further studies are required to elucidate the detailed mechanism.

Skin inflammation is strongly involved in the pathogenesis of inflammatory skin diseases^49^. Indeed, previous studies have shown that the inhibition of phosphodiesterase 4 (PDE4), a cAMP-degrading enzyme, improves atopic dermatitis and psoriasis^50,51^; consequently, certain PDE4 inhibitors have been approved to treat these diseases^52,53^. These clinical results suggest that cAMP plays a key role in the pathogenesis of inflammatory skin diseases. Since CO_2_ has been shown to promote cAMP production in keratinocytes, it may exert a similar, albeit mild, effect as PDE4 inhibitors. Thus, the topical application of CO_2_ may serve as a novel therapeutic approach for treating patients with inflammatory skin disorders.

In conclusion, in the present study, it was demonstrated that CO_2_ activates GPR65 via extracellular acidification and exerts anti-inflammatory effects by suppressing NF-κB activation in keratinocytes. Moreover, the topical application of a high-CO_2_ concentration formulation inhibited UVB-induced erythema formation, implying that CO_2_ suppresses skin inflammation in vivo. Therefore, our hypothesis, derived from the obtained results, states that CO_2_ is a unique gas molecule that can suppress skin inflammation.

## Methods

### High-CO_2_ concentration formulation

The formulations used in the present study were prepared as described previously^25^. The high-CO_2_ concentration and control formulations used the same base composition; however, the high-CO_2_ concentration formulation contained ~ 1500–2000 ppm of CO_2_ in the form of microbubbles.

### Cell cultures

HEKn (Invitrogen, Carlsbad, CA, USA) isolated from the foreskin of neonatal Caucasian subjects was cultured in EpiLife medium (Gibco, Waltham, MA, USA) supplemented with additive agents (HuMedia-KG kit; KURABO, Osaka, Japan). Before the experiments, the culture medium was replaced with fresh medium without additive agents. For the high-CO_2_ experiments, cells were cultured in a humidified incubator under 15% CO_2_ for 24 h. For the low-pH experiments, cells were cultured in a pH-modified culture medium for 24 h, with 1 M HCl and 1 M NaOH used to adjust the pH of the culture medium. For dibutyryl cAMP (dbcAMP; Sigma-Aldrich, St. Louis, MO, USA) treatment, cells were incubated with 0.1, 0.2, and 0.5 mM of dbcAMP for 24 h.

A three-dimensional (3D) epidermis (LabCyte EPI-MODEL 12; Japan Tissue Engineering Co. Ltd, Aichi, Japan) was cultured and maintained according to the manufacturer's instructions. For the high-CO_2_ experiments, high-CO_2_ concentration and control formulations were applied to the surface of the 3D epidermis for 12 h. Next, the cells and 3D epidermis were washed with Dulbecco’s phosphate buffered saline (DPBS; Gibco) and exposed to 20 mJ/cm^2^ UVB using a BIO-UV EXPOSURE instrument (SEN LIGHTS Co. Ltd, Osaka, Japan).

### Intra-epidermal pH imaging

For intra-epidermal pH imaging, multiphoton laser microscopy (DermaInspect; JenLab, Jena, Germany) was applied. Since the fluorescence lifetime (FT) of 2′,7′-bis(carboxyethyl)-4 or 5-carboxyfluorescein (BCECF) has been shown to correlate with pH^54^, the FT was used as an indicator of intra-epidermal pH. The culture medium of the 3D epidermis was replaced with Hanks’ balanced salt solution (HBSS; Gibco) supplemented with BCECF or BCECF-AM (DOJINDO, Kumamoto, Japan) at a final concentration of 10 μM. After 15 min, the 3D epidermis was washed with HBSS solution and used for pH imaging, whereby 3–5 mW of laser light was focused on to the epidermis from 10 μm above the insert membrane. Fluorescent images were acquired inside the epidermis and analyzed using SPC Image 2.9.4 (Becker & Hickl GmbH, Berlin, Germany). A bi-exponential fit was used on the fluorescence decay profiles, and the FT was determined for each pixel, with the largest value recorded as the representative FT of the image.

### G protein-coupled receptor (GPCR) expression measurement

To measure GPCR expression, HEKn were grown to 50% confluence and collected for RNA extraction. Digital PCR was performed on a QuantStudio 3D Digital PCR System platform consisting of a ProFlex PCR machine (including a chip adapter kit), an automatic chip loader, and a QuantStudio 3D Instrument (Life Technologies, Carlsbad, CA, USA). Specific TaqMan probes for digital PCR (Supplementary Table 1; see Supplementary Information) were selected from the TaqMan Gene Expression Assay (Applied Biosystems, Foster City, CA, USA).

### siRNA experiments

HEKn were grown to 50% confluence and transfected with specific siRNA against GPR65 or control non-target siRNA (Dharmacon, Lafayette, CO, USA) using Lipofectamine RNAiMAX transfection reagent (Invitrogen, Carlsbad, CA, USA) according to the manufacturer’s instructions. After 48 h, the cells were used for the high-CO_2_ experiments.

### cAMP measurement

HEKn transfected with siRNA for 48 h were incubated under 15% CO_2_ for 24 h in the presence of the phosphodiesterase inhibitor 0.5 mM 3-isobutyl-1-methylxanthine (Sigma-Aldrich, St. Louis, MO, USA) and then collected. Intracellular cAMP concentration was measured using a cAMP-Glo Assay (Promega, Madison, WI, USA) according to the manufacturer’s instructions.

### RNA isolation and qRT-PCR

HEKn and the 3D epidermis were collected 8 h after UVB exposure. Total RNA was isolated using an RNeasy Mini Kit (Qiagen, Hilden, Germany) reverse-transcribed into cDNA using a High Capacity RNA-to-cDNA Kit (Applied Biosystems). Quantitative real-time PCR (qRT-PCR) was performed using a TaqMan Gene Expression Assay (Applied Biosystems) with specific probes for each target gene (Supplementary Table 2; see Supplementary Information). Results were normalized to RPLP0.

### Enzyme-linked immunosorbent assay (ELISA)

TNFα and IL-6 concentrations in the culture media of HEKn were measured using cytokine ELISA kits (Diaclone, Besancon, France) according to the manufacturer’s instructions. Absorbance was measured using an SH-9000 Lab microplate reader (Corona Electric, Ibaragi, Japan).

### Western blotting

For whole-cell protein extraction, HEKn were treated with radio-immunoprecipitation assay (RIPA) lysis buffer (Thermo Fisher Scientific, San Jose, CA, USA) supplemented with a protease/phosphatase inhibitor cocktail (Cell Signaling Technology, Beverly, MA, USA). For nuclear and cytoplasmic extraction, a nuclear extraction kit (Active Motif, Carlsbad, CA, USA) was used according to the manufacturer’s instructions. Protein was quantified in each lysate using a BCA protein assay kit (Thermo Fisher Scientific), and lysates containing equal amounts of proteins were loaded onto Mini-PROTEAN TGX gels (Bio-Rad, Hercules, CA, USA), electrophoresed, and transferred to 0.2-μm polyvinylidene fluoride (PVDF) membranes using a Trans-Blot Turbo System (Bio-Rad). The membranes were blocked with PVDF Blocking Reagent (TOYOBO, Osaka, Japan) and incubated with primary and horseradish peroxidase (HRP)-conjugated secondary antibodies (Supplementary Table 3; see Supplementary Information). Bound antibodies were detected using ECL Prime Western Blotting Detection Reagent (GE Healthcare, Arlington Heights, IL, USA).

### Human study

The present study was approved by the Ethical Committee of Kao Corporation and conducted in accordance with the study protocol, ethical guidelines for clinical research, and ethical principles based on the Helsinki Declaration; it was registered with the UMIN Clinical Trials Registration System and is publicly available (# UMIN000019152). All study participants were informed regarding the content matter of the present study, and they provided informed consent. A total of nine healthy Japanese men between 20 and 50 years were recruited for this double-blind, placebo-controlled study. High-CO_2_ and control formulations were applied to a designated area (5 × 15 cm) on the inside of the left upper arm twice daily (morning and night). After 2 weeks, each site was irradiated with 10, 20, 30, 40, 50, 60, or 70 mJ/cm^2^ of UVB (light source: UV-B lamp, GL20SE; Sankyo Denki, Kanagawa, Japan). The following day, minimal erythema dose (MED) was judged, and color was measured using a spectrocolorimeter to confirm erythema formation. The *a value of the L*a*b* colorimetric system was used as an index of erythema.

### Statistical analysis

Data are presented as the mean ± SEM. For the experiments using HEKn and 3D epidermis, between-group differences were analyzed by the unpaired Student’s *t* tests, Dunnett’s test, or Tukey–Kramer multiple comparison tests, as appropriate. For the human study, between-group differences were analyzed by the paired Student’s *t* tests.

## Supplementary Information

Supplementary Information.

## Data Availability

The datasets generated during and/or analyzed during the current study are available from the corresponding author on reasonable request.
