# Supplementary material for: Carbon dioxide inhibits UVB-induced inflammatory response by activating the proton-sensing receptor, GPR65, in human keratinocytes
Source: Sci Rep. 2021 Jan 11;11:379. doi: 10.1038/s41598-020-79519-0 (PMC7801444; doi:10.1038/s41598-020-79519-0)
Supplement: Supplementary file 1 — Supplementary Information. [file 41598_2020_79519_MOESM1_ESM.pdf]

Supplementary Information

Carbon dioxide inhibits UVB-induced inflammatory response by activating the proton-sensing receptor, GPR65, in human keratinocytes

Keimon Sayama[1, 2], Katsuyuki Yuki[1], Keiichi Sugata[1], Satoko Fukagawa[1], Tetsuji Yamamoto[1], Shigaku Ikeda[2], Takatoshi Murase[1]*

[1] Biological Science Research, Kao Corporation, Tochigi, Japan

[2] Department of Dermatology and Allergology, Juntendo University Graduate School of Medicine, Tokyo, Japan.

* Corresponding author

Takatoshi Murase, 2606, Akabane, Ichikai-machi, Haga-gun, Tochigi, 321-3497, Japan

E-mail: murase.takatoshi@kao.com

Legends to Supplementary Figures

Supplementary Figure 1.
Full length western blot luminescent signals for IκBα (a) and αTubulin (b) relating to cropped data presentated in Fig. 3e

Supplementary Figure 2.
Full length western blot luminescent signals for p65 (a), Lamin A/C (b) and αTubulin (c) relating to cropped data presentated in Fig. 3f

Supplementary Figure 3.
Full length western blot luminescent signals for P-p38 (a), p38 (b), P-ERK1/2 (c), ERK1/2 (d), P-SAPK/JNK (e) and SAPK/JNK (f) relating to cropped data presentated in Fig. 3g

Supplementary Figure 4.
Full length western blot membrane images for IκBα (a) and αTubulin (b) relating to cropped data presentated in Fig. 3e

Supplementary Figure 5.
Full length western blot membrane images for p65 (a), Lamin A/C (b) and αTubulin (c) relating to cropped data presentated in Fig. 3f

Supplementary Figure 6.
Full length western blot membrane images for P-p38 (a), p38 (b), P-ERK1/2 (c), ERK1/2 (d), P-SAPK/JNK (e) and SAPK/JNK (f) relating to cropped data presentated in Fig. 3g

Supplementary Table 1. Taqman probes used in Digital PCR.

Gene Symbol	Gene name	Assay ID	Company
**GPR4**	G protein-coupled receptor 4	Hs00269247_s1	Thermo Fisher Scientific
**GPR65**	G protein-coupled receptor 65	Hs00268858_s1	Thermo Fisher Scientific
**GPR68**	G protein-coupled receptor 68	Hs01871869_s1	Thermo Fisher Scientific
**GPR132**	G protein-coupled receptor 132	Hs01871869_s1	Thermo Fisher Scientific

Supplementary Table 2. Taqman probes used in qRT-PCR.

Gene Symbol	Gene name	Assay ID	Company
**RPLP0**	ribosomal protein, large, P0	Hs99999902_m1	Thermo Fisher Scientific
**TNF**	tumor necrosis factor	Hs01113624_g1	Thermo Fisher Scientific
**IL-6**	interleukin 6	Hs00985639_m1	Thermo Fisher Scientific

Supplementary Table 3. Antibodies used in the present study.

Primary antibodies	CST code	Company	Dilution
**αTubulin**	2144S	Cell Signaling Technology	1:1000
**Lamin A/C**	4777S	Cell Signaling Technology	1:1000
**p65**	8242S	Cell Signaling Technology	1:1000
**I-κBα**	4814S	Cell Signaling Technology	1:1000
**p38**	9212S	Cell Signaling Technology	1:1000
**P-p38**	4511S	Cell Signaling Technology	1:1000
**SAPK/JNK**	9252S	Cell Signaling Technology	1:1000
**P-SAPK/JNK**	4671S	Cell Signaling Technology	1:1000
**Erk1/2**	4348S	Cell Signaling Technology	1:1000
**P-Erk1/2**	8544S	Cell Signaling Technology	1:1000
**Secondary antibodies**	**CST code**	**Company**	**Dilution**
**anti-rabbit IgG**	7074S	Cell Signaling Technology	1:5000
**anti-mouse IgG**	7076S	Cell Signaling Technology	1:5000

(a)

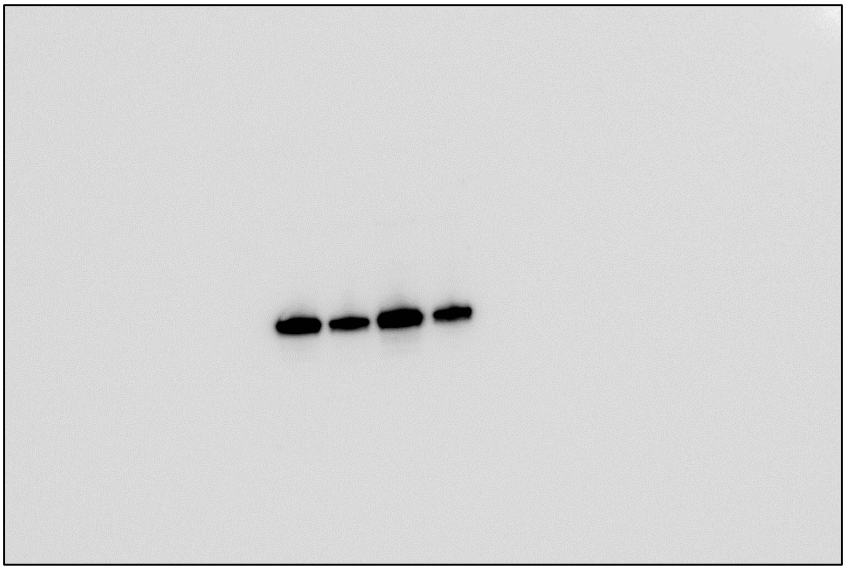

(b)

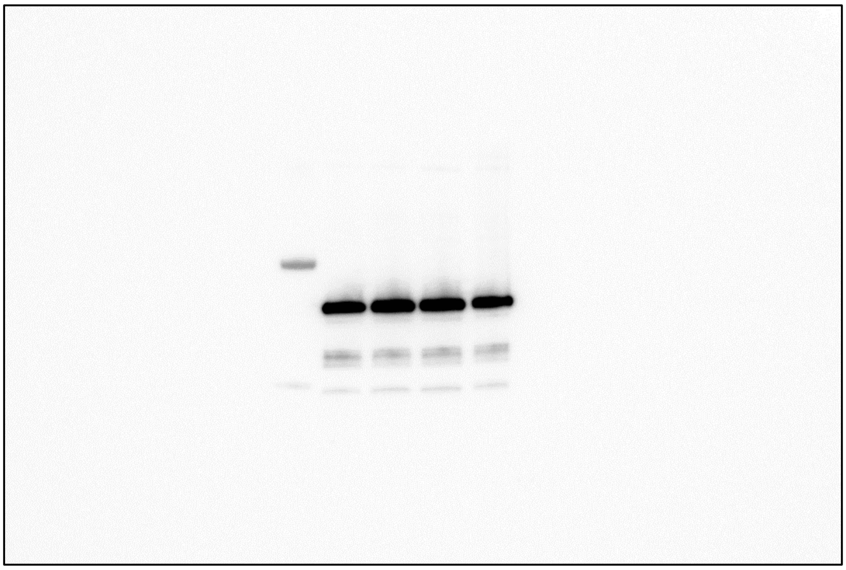

Supplementary Figure 1.

(a)
(b)
(c)

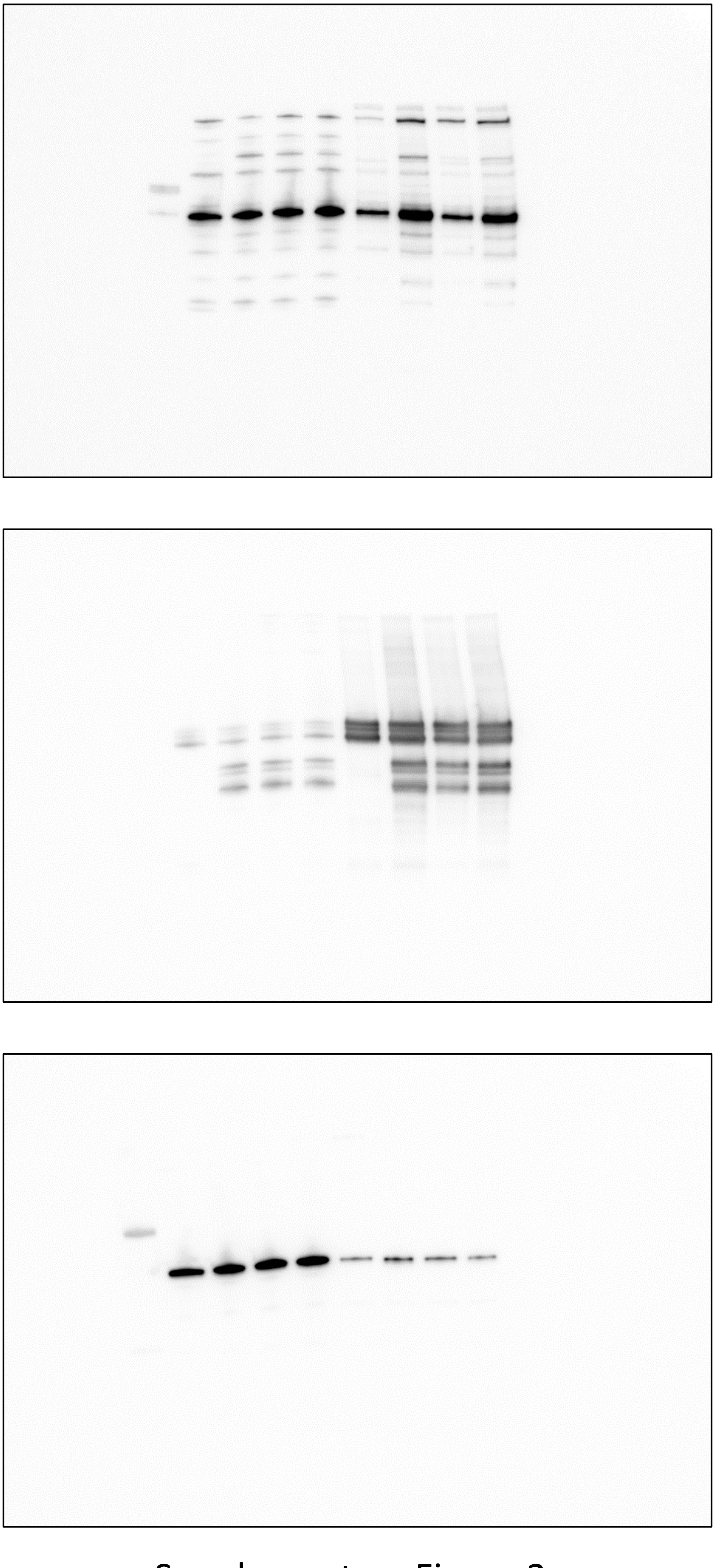

Supplementary Figure 2.

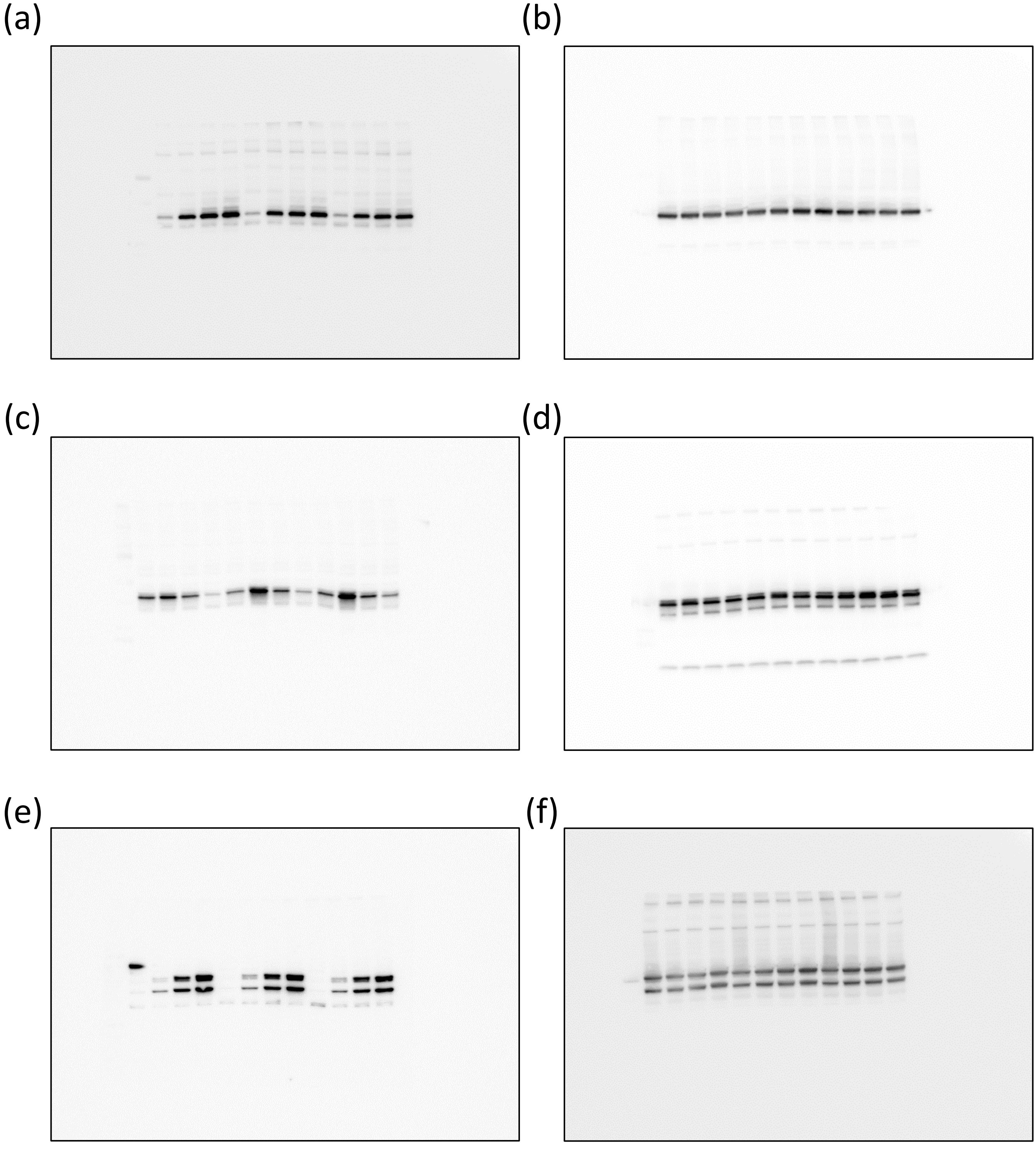

Supplementary Figure 3.

(a)

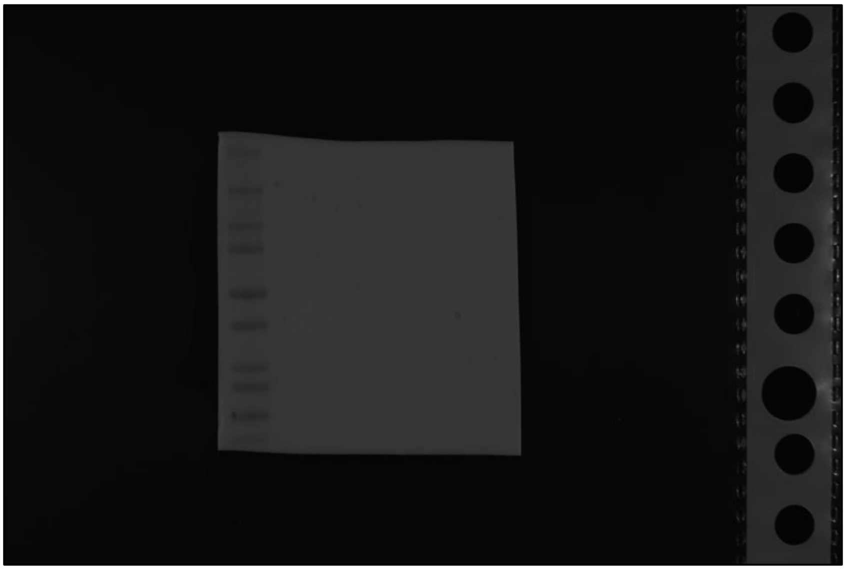

(b)

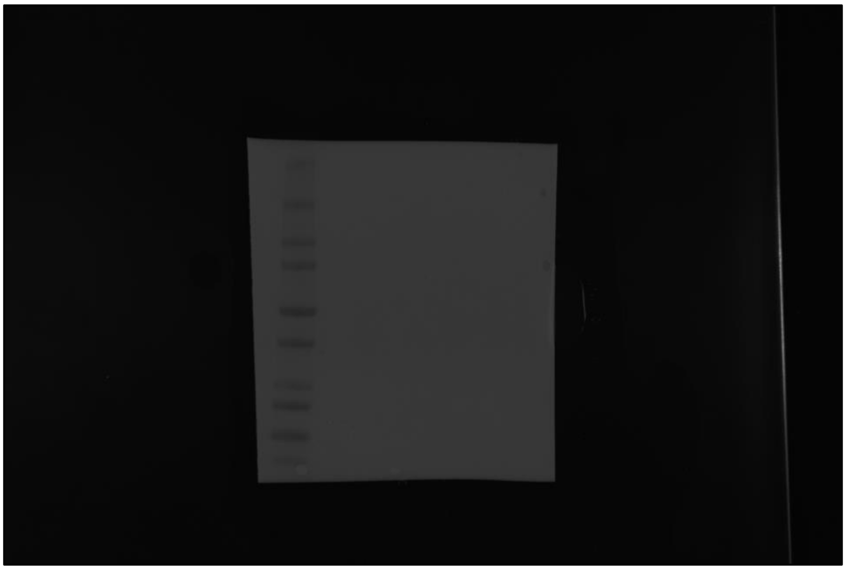

Supplementary Figure 4.

(a)
(b)
(c)
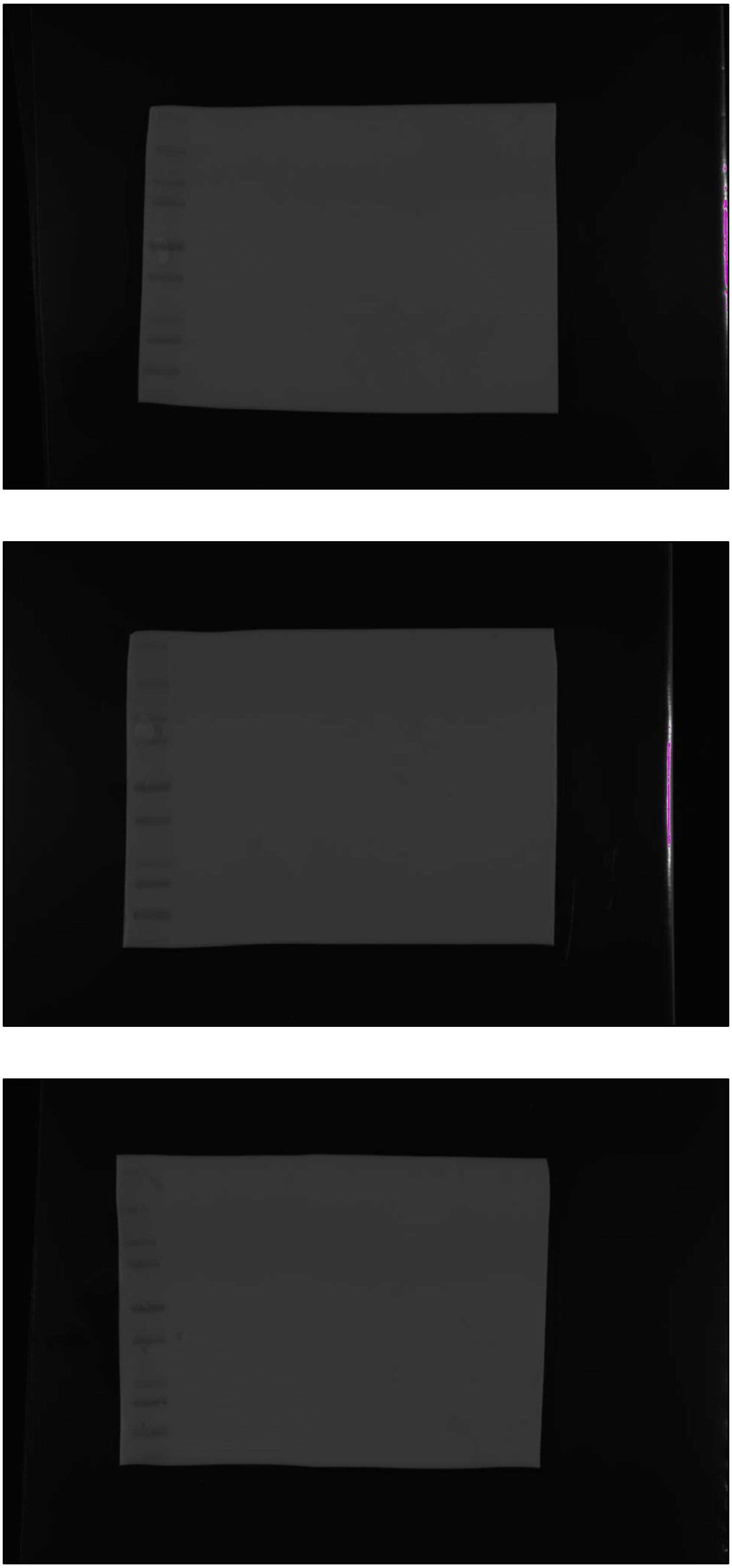

Supplementary Figure 5.

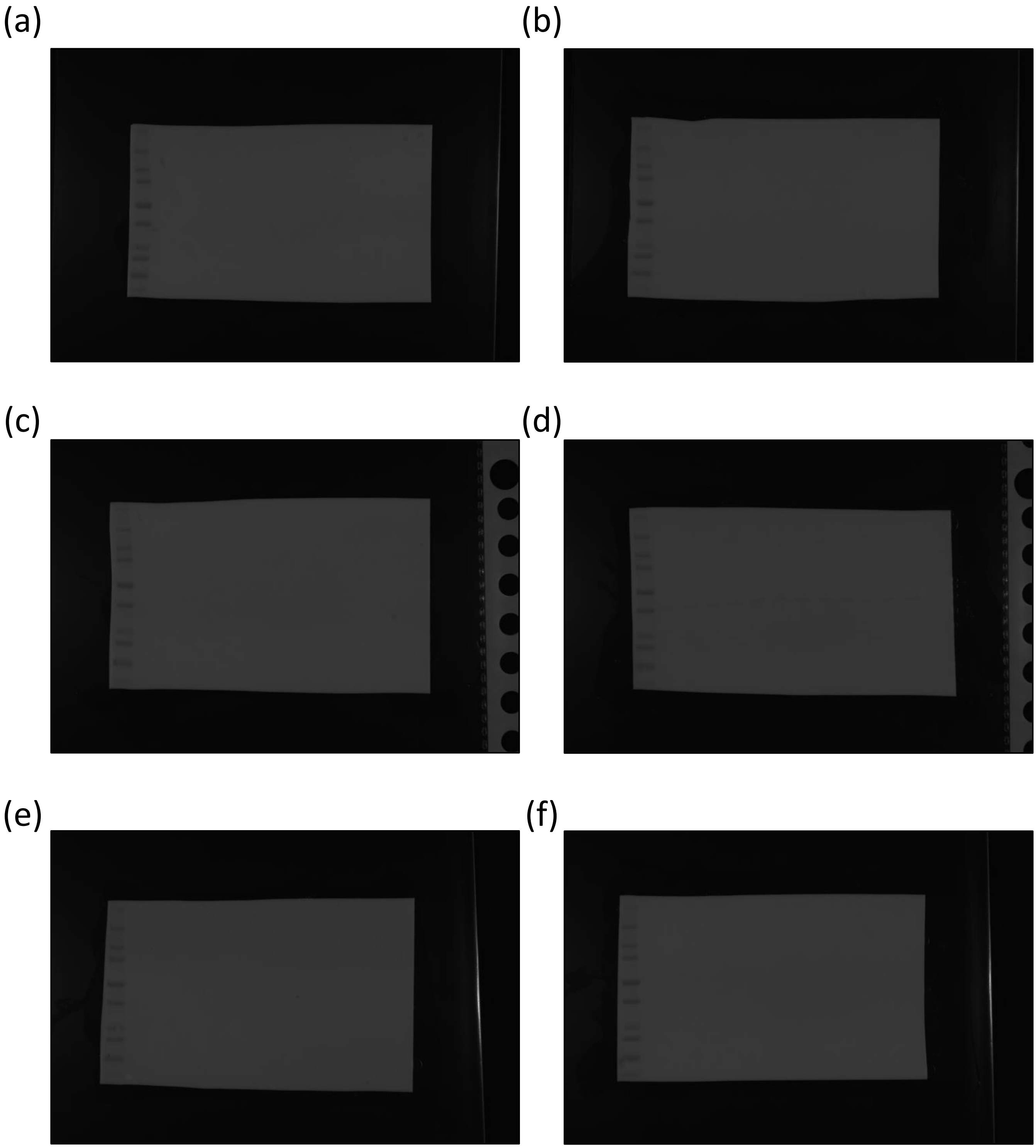

Supplementary Figure 6.
